# 195. Intravenous to Oral Antibiotics Versus Intravenous Antibiotics: A Step-Up or a Step-Down for Extended Spectrum Beta-Lactamase Producing Urinary Tract Infections?

**DOI:** 10.1093/ofid/ofab466.195

**Published:** 2021-12-04

**Authors:** Kelly C Gamble, Dusten T Rose, Julia Sapozhnikov

**Affiliations:** 1 Ascension Seton, Austin, TX; 2 Seton Healthcare Family, Austin, TX; 3 Ascension Texas- Dell Children’s Medical Center of Central Texas, Austin, Texas

## Abstract

**Background:**

The treatment of extended-spectrum beta-lactamase (ESBL)-producing urinary tract infections (UTI) may include either intravenous (IV) or oral (PO) antibiotics, according to the Infectious Diseases Society of America guidelines for resistant gram negative infections. The purpose of this study is to evaluate if PO step-down antibiotics, the switch group, compared to continued IV therapy in these UTIs affects clinical outcomes.

**Methods:**

This multicenter retrospective cohort study was conducted in hospitalized patients with an ESBL-producing UTI between July 2016 and March 2020. The control group received a complete antibiotic course with a carbapenem. The switch group was transitioned to an oral agent within five days from initiation of a carbapenem. The primary endpoint was a composite all-cause clinical failure, which was defined as readmission or hospital mortality within 30 days of hospital discharge or a change in antibiotic during hospital admission. The secondary endpoints included individual components of the primary outcome, readmission indication, inpatient length of stay, direct antibiotic costs, and adverse events.

**Results:**

The study included 153 patients: 95 and 58 patients in the control and switch groups, respectively. Demographics between the two groups were similar (Table 1). The mean ± SD duration of therapy was 8.7 ± 3.1 and 7.1 ± 3.3 days, respectively. Four oral agents were used for step-down therapy (Figure 1). The primary outcome occurred in 28% in both groups (27 vs 16 patients, p=0.91). The individual components of the primary outcome and readmission indication were also similar: readmission (93% vs 94%, p=0.95), readmission due to a recurrent UTI (33% vs 25%, p=0.73), hospital mortality (7% vs 6%, p=1.0), and change in antibiotic (0% vs 2%, p=0.38). The median (IQR) length of stay and direct antibiotic cost in the control and switch groups were 8 (6) vs 5 (2) days (p< 0.01) and &278 (&244) vs &180 (&104) (p< 0.01), respectively. Adverse events were similar in both groups except for diarrhea (15% vs 2%, p=0.01).

Table 1. Baseline Demographics. SD: standard deviation, ICU: intensive care unit, qSOFA: quick Sequential Organ Failure Assessment, ESBL: extended spectrum beta-lactamase, UTI: urinary tract infection

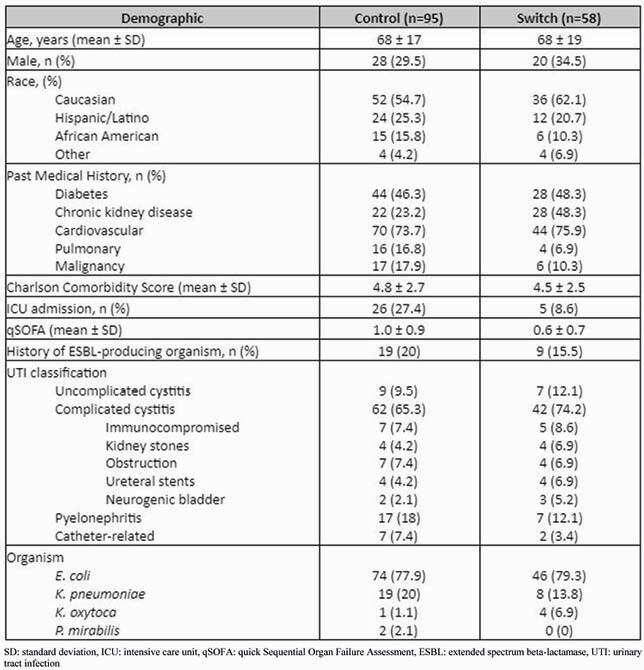

Figure 1. Oral Antibiotics. QD: once daily, BID: twice daily, Q2D: every 2 days, Q3D: every 3 days, DS tab: double strength tablet

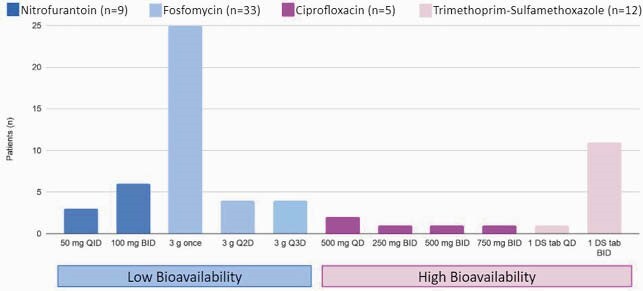

**Conclusion:**

There was no difference in clinical failure, readmission rate, mortality rate, or change in antibiotic between the control and switch groups; however, the switch group was associated with reduced hospital length of stay and direct antibiotic cost.

**Disclosures:**

**All Authors**: No reported disclosures

